# Temporal trends of dialysis requiring acute kidney injury after orthotopic cardiac and liver transplant hospitalizations

**DOI:** 10.1186/s12882-017-0657-8

**Published:** 2017-07-19

**Authors:** Girish N. Nadkarni, Kinsuk Chauhan, Achint Patel, Aparna Saha, Priti Poojary, Sunil Kamat, Shanti Patel, Rocco Ferrandino, Ioannis Konstantinidis, Pranav S. Garimella, Madhav C. Menon, Charuhas V. Thakar

**Affiliations:** 1 Division of Nephrology, Department of Medicine, Icahn School of Medicine at Mount Sinai, New York, NY USA; 2 Division of Critical Care, Department of Medicine, Sir H.N. Reliance Hospital and Research Center, Mumbai, India; 3 Division of Nephrology, Department of Medicine, University of California San Diego, San Diego, CA USA; 4 Division of Nephrology, Kidney CARE Program, University of Cincinnati, Cincinnati, USA; 5 Renal Section, Cincinnati VA Medical Center, Cincinnati, OH USA; 6 Division of Nephrology and Hypertension, ML 0585, 231 Albert B Sabin Way, Cincinnati, OH 45267 USA

**Keywords:** Acute kidney injury, Cardiac transplant, Liver transplant, Mortality, Outcomes

## Abstract

**Background:**

The epidemiology and outcomes of acute kidney injury (AKI) in prevalent non-renal solid organ transplant recipients is unknown.

**Methods:**

We assessed the epidemiology of trends in acute kidney injury (AKI) in orthotopic cardiac and liver transplant recipients in the United States. We used the Nationwide Inpatient Sample to evaluate the yearly incidence trends (2002 to 2013) of the primary outcome, defined as AKI requiring dialysis (AKI-D) in hospitalizations after cardiac and liver transplantation. We also evaluated the trend and impact of AKI-D on hospital mortality and adverse discharge using adjusted odds ratios (aOR).

**Results:**

The proportion of hospitalizations with AKI (9.7 to 32.7% in cardiac and 8.5 to 28.1% in liver transplant hospitalizations; p_trend_<0.01) and AKI-D (1.63 to 2.33% in cardiac and 1.32 to 2.65% in liver transplant hospitalizations; p_trend_<0.01) increased from 2002-2013. This increase in AKI-D was explained by changes in race and increase in age and comorbidity burden of transplant hospitalizations. AKI-D was associated with increased odds of in hospital mortality (aOR 2.85; 95% CI 2.11-3.80 in cardiac and aOR 2.00; 95% CI 1.55-2.59 in liver transplant hospitalizations) and adverse discharge [discharge other than home] (aOR 1.97; 95% CI 1.53-2.55 in cardiac and 1.91; 95% CI 1.57-2.30 in liver transplant hospitalizations).

**Conclusions:**

This study highlights the growing burden of AKI-D in non-renal solid organ transplant recipients and its devastating impact, and emphasizes the need to develop strategies to reduce the risk of AKI to improve health outcomes.

**Electronic supplementary material:**

The online version of this article (doi:10.1186/s12882-017-0657-8) contains supplementary material, which is available to authorized users.

## Background

Acute kidney injury (AKI) is an common condition in hospitalized patients [1], and is associated with high morbidity and mortality [2–4]. Approximately 1-in-3 cases of AKI occur in peri-operative settings, including non renal solid-organ transplantation [5]. The incidence of AKI has been increasing in hospitalized patients [6, 7]. The sequelae of AKI include poor long-term survival, increased risk of re-admissions, worsening of CKD, and progression to end-stage renal disease; taken together, this adversely impacts both, health outcomes as well as health care resources.

The incidence of non-renal solid organ transplantation (NRSOT) including cardiac and liver transplantation has been increasing in the United States [8]. NRSOT recipients are at risk of AKI in the short-term, and progressive loss of kidney function in the long-term, leading to end-stage renal disease (ESRD) [9]. The incidence of AKI after cardiac transplant and liver transplant can range between 10 and 50% [10, 11]. Risk of AKI in the short-term is primarily dependent on co-morbid conditions and post-operative complications (e.g. sepsis) associated with acute care hospitalizations. On the other hand, long-term progressive loss of renal function is associated with multitude of factors, including exposure to calcineurin-inhibitor use and allograft dysfunction.

Overall, allograft and patient survival have been improving over time, and this may be reflective of our ability to provide better supportive care [12–14]. With the advent of improved induction and mantainence immunosuppressive therapies there have been changes in both the timing of initiation as well as dose/duration of calcineurin inhibitor use in the post-transplant period. On the other hand, changes in co-morbid characteristics (e.g. donor/recipient criteria) may also affect the risk of post-operative complications. Thus, prospect of declining renal function, comorbid disease burden, and reasons for acute care hospitalizations, all contribute towards a pre-disposition to AKI in transplant recipients.

There are several (mostly single center) studies outlining frequency, risk factors or outcomes of AKI in immediate post-transplant period [10, 15]. However, there are limited data on the epidemiology of trends in AKI over time, particularly severe AKI requiring dialysis (AKI-D), in all hospitalizations among orthotopic cardiac and liver transplant recipients. We hypothesized that changes in treatment regimens, co-morbid characteristics, and post-operative complications will influence the risk of AKI during hospitalization over time. We tested this clinical hypothesis by utilizing a large, nationally representative database to explore national trends and impact of AKI in orthotopic cardiac and liver transplant hospitalizations.

## Methods

### Data sources

We extracted our study cohort from the Nationwide Inpatient Sample (NIS) and National Inpatient Sample of Healthcare Cost and Utilization Project (HCUP), Agency for Healthcare Research and Quality [16]. We selected the time period 2002–2013 based on availability of complete data. Since the data are completely deidentified and publically available with a data use agreement from the HCUP, the study was considered to be Institutional Review Board exempt by the Mount Sinai Institutional Review Board.

### Study population and design

This study was a retrospective database analysis utilizing the Nationwide Inpatient Sample database from 2002–2013. We included adult hospitalizations (age>18 years of age) with prevalent cardiac and liver transplant status by the International Classification of Diseases, Ninth Revision, Clinical Modification (ICD-9-CM) codes V42.1 and V42.7 respectively. We excluded other solid organ transplants (lung, bowel) due to lack of adequate sample size for modeling and excluded pancreas transplant due to high concurrence with kidney transplants. We also excluded hospitalizations in the immediate postoperative period and hospitalizations for complications of transplant using ICD-9-CM codes of 996.8 in any diagnosis field and hospitalizations with transplant related procedures using Clinical Classification Software (CCS) for ICD-9-CM categories 13, 64, 105 and 176. To avoid misclassification of hospitalizations for chronic hemodialysis initiation, we excluded those with procedure codes for arteriovenous access creation or revision [3]. Similarly, we excluded hospitalizations with dialysis codes but no AKI code, assuming that patients were receiving dialysis for ESRD. We defined AKI by ICD-9-CM code 584.xx and dialysis procedure was identified by presence of ICD-9-CM procedure code of 39.95 or diagnosis code of v45.11, v56.0 or v56.1 [17]. This approach has been used previously and has sensitivity of 90.4%, specificity of 93.8%, and positive and negative predictive value of 94.0% and 90.0%, respectively [17].

### Definition of variables

We extracted baseline characteristics of the study population. Patient-level characteristics included age, gender, race, quartile classification of median household income extrapolated from ZIP Code, and primary payer (Medicare/Medicaid, private insurance, self-pay, or no charge). Hospital-level characteristics included hospital location307 (urban/rural), hospital bed size (small, medium, and large) and teaching status. We defined the severity of co-morbid conditions using Deyo’s modification of the Charlson co-morbidity index (CCI), which contains 17 co-morbid conditions with differential weights.

We classified AKI during hospitalization in two different ways; AKI with dialysis (AKI-D) and AKI without dialysis, defined based on the descriptions provided above. Discharge disposition was grouped by [1] home or short-term facility (routine, short-term hospital, home intravenous provider, another rehabilitation facility, another institution for outpatient services, institution for outpatient services, discharged alive, destination unknown) or [2] adverse discharge (skilled nursing facility, intermediate care, hospice home, hospice medical facility, long-term care hospital, certified nursing facility). This dichotomization of discharge disposition is commonly used in studies utilizing NIS data [18].

### Statistical analysis

Although we reported temporal trends for all AKI, we present detailed results for only AKI-D. We chose to do this because the validity of administrative codes for non-dialysis requiring AKI are poor and have changed over time, limiting their accuracy [19]. We compared the baseline characteristics of NRSOT hospitalizations in two groups: no AKI-D and AKI-D. We utilized the chi-square test for categorical variables, Student’s t-test for normally distributed continuous variables, and the Wilcoxon rank-sum test for non-normally distributed continuous variables. We utilized survey logistic regression models to estimate the impact of AKI-D on mortality and discharge disposition. Survey logistic regression modeling is an appropriate analysis for data with nested observations such as the NIS, which is stratified in clusters to produce national estimates. We constructed final models after adjusting for confounders, testing for potential interactions, and ensuring no multi-co-linearity between predictor variables. We also performed a secondary survey regression analysis to explore potential reasons for temporal changes in AKI-D. In the first model we included only calendar year as the predictor and AKI-D as the outcome. Additional patient level covariates were added in the second model to determine the degree to which they explained temporal trends. Finally, concurrent acute/chronic comorbidities and procedures that are known risk factors for AKI-D were included in a third model. We performed all association and trend analysis using designated weight values to produce nationally representative estimates. A two-tailed p value ≤ 0.01 using Bonferroni correction for multiple testing was considered statistically significant. We utilized SAS 9.3 (SAS Institute Inc. Cary, North Carolina) for all analyses.

## Results

We identified 130,143 hospitalizations with cardiac transplant from 2002-13 of which 30744 (23.7%) had AKI and 2776 (2.13%) had AKI-D. Similarly, we identified 266,987 hospitalizations with liver transplant of which 56324 (21.1%) had AKI and 5689 (2.14%) had AKI-D (Additional file 1: Figure S1).

**Fig. 1:**
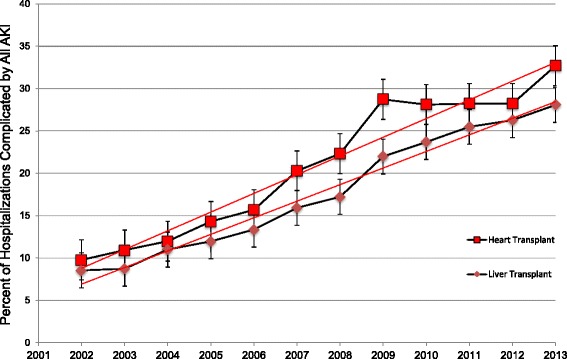
Temporal Trends of Orthotopic Heart and Liver Transplant Hospitalizations Complicated by All Acute Kidney Injury. This figure demonstrates the proportion of orthotopic heart (*red squares*) and liver (*maroon diamonds*) complicated by acute kidney injury per year. The error bars around the estimates indicate standard errors. The *red lines* indicate trends in the proportions from 2002 to 2013

### Temporal trends of AKI in cardiac and liver transplant hospitalizations

**Fig. 2:**
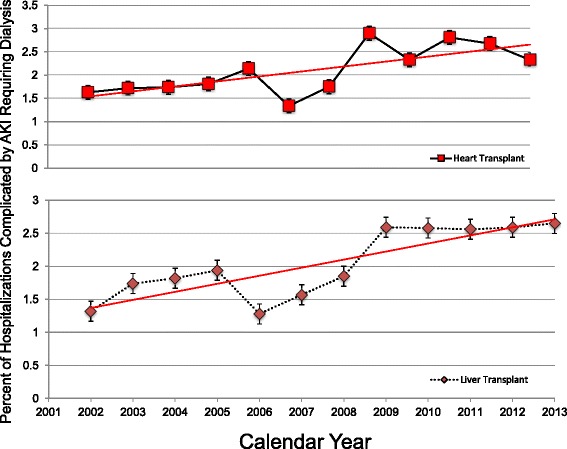
Temporal Trends of Orthotopic Heart and Liver Transplant Hospitalizations Complicated by Acute Kidney Injury Requiring Dialysis. This figure demonstrates the proportion of orthotopic heart (*red squares*) and liver (*maroon diamonds*) complicated by acute kidney injury requiring dialysis per year. The error bars around the estimates indicate standard errors. The *red lines* indicate trends in the proportions from 2002 to 2013

As shown in Fig. 1, the percentage of hospitalizations complicated by AKI in both cardiac and liver transplant hospitalizations tripled over the study period from 2002 to 2013 (9.7% to 32.7% in cardiac; p_trend_<0.01 and 8.5% to 28.1% in liver transplant hospitalizations; p_trend_<0.01). Analysis of annual rates demonstrated a forty five percent increase in AKI-D in cardiac transplant admissions over the study period, from 1.63% in 2002 to 2.33% hospitalizations in 2013; p_trend_<0.01. Similarly, we demonstrated a two-fold increase in AKI-D in liver transplant admissions over the study period, from 1.32% in 2002 to 2.65% hospitalizations in 2013; p_trend_<0.01. (Fig. 2) Subgroup analysis by age showed that this increase was particularly striking in the age group >65 years with an increase from 1.2% to 2.8% in heart transplant and from 1.6 to 3.2% in liver transplant hospitalizations (Additional file 2: Table S1).

**Table 1:** Baseline characteristics of study population stratified by acute kidney injury requiring dialysis

	Heart Transplant Without AKI-D (*n*=127367)	Heart Transplant With AKI-D (*n*=2776)	*p*	Liver Transplant Without AKI-D (*n*=261298)	Liver Transplant With AKI-D (*n*=5689)	*p*
Age Mean (SE)	58.2 (0.25)	60.7 (0.65)	<0.01	55.6 (0.16)	58.7 (0.38)	<0.01
18–34	8.75	6.79		8.21	4.06	
35–49	12.46	8.75		15.69	11.21	
50–64	40.1	38.09		51.9	54.07	
≥65	38.7	46.37		24.2	30.65	
Gender			<0.01			<0.01
Male	72.55	76.43		59.88	63.14	
Female	27.41	23.57		40.04	36.86	
Race			0.05			<0.01
White	63.34	66.19		61.73	60.62	
Black	11.38	11.96		6.53	8.1	
Hispanic	5.33	5.13		10.79	11.76	
Others	3.51	4.62		5.28	5.89	
Missing	16.44	12.1		15.67	13.63	
Charlson Comorbidity Index			<0.01			<0.01
0	27.43	12.56		34.77	12.33	
1	22.56	12.77		24.11	11.17	
2	50	74.67		41.11	76.49	
Concurrent Diagnosis						
Diabetes Mellitus	41.04	37.09	<0.01	39.96	41.35	0.03
Hypertension	63.96	74.18	<0.01	49.29	65.21	<0.01
Chronic Kidney Disease	31.26	31.75	0.57	20.7	25.07	<0.01
Acute or Chronic Liver Disease	3.42	13.22	<0.01	NA	NA	NA
Liver or intrahepatic biliary Cancer	0.09	0	0.111	6.97	3.94	<0.01
Sepsis	6.33	31.25	<0.01	7	27.52	<0.01
Acute Myocardial Infarction	0.68	1.37	<0.01	1.66	3.84	<0.01
Primary Acute Heart Failure	2.92	3.28	0.2684	1.94	4.68	<0.001
Cardiac catheterizations	4.73	2.26	<0.01	2.77	2.47	0.1707
Mechanical ventilation	4.73	2.26	<0.01	2.8	22.51	<0.01
Zip code Income (%)			<0.01			<0.01
0-25 percentile	22.01	19.27		22.72	23.67	
26-50 percentile	23.46	22.12		23.08	21.62	
51-75 percentile	23.7	26.76		23.87	25.11	
76-100 percentile	22.99	25.47		22.77	23.56	
Primary Payer			<0.01			<0.01
Medicare/Medicaid	68	73.47		59.5	61.61	
Private	28.54	25.33		36.53	34.87	
Uninsured/Self pay	3.33	1.19		3.81	3.52	
Hospital Characteristics						
Hospital bed size			<0.01			<0.01
Small	5.27	3.07		7.67	6.43	
Medium	15.58	14.21		17.58	14.9	
Large	78.83	82.55		74.16	77.86	
Hospital Location			<0.01			<0.01
Rural	6.35	0.82		6.63	1.94	
Urban non teaching	21.35	21.96		24.08	22.5	
Urban teaching	71.97	77.06		68.68	74.75	

Both populations were compared utilizing chi-square test, Wilcoxon rank sum test and survey regression depending on the distributions of individual variables. ¥ Quartile classification of the estimated median household income of residents in the patient’s ZIP Code. These values are derived from ZIP Code-demographic data obtained from Claritas

#### Cardiac transplant hospitalizations

**Fig. 3:**
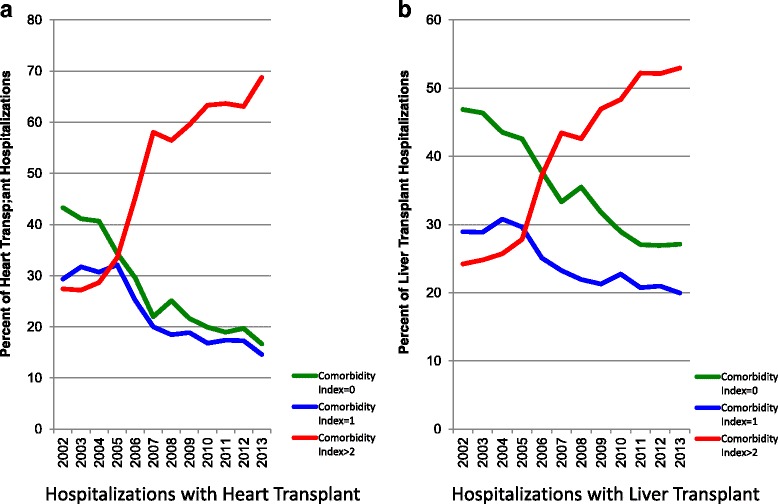
Temporal Trends in comorbidity index in all Orthotopic Heart and Liver Transplant Hospitalizations. This figure demonstrates the percentage of Orthotopic Heart (**a**) and Liver Transplant Hospitalizations (**b**) with different classes of Charlson comorbidity index. As shown, the percentage of orthotopic heart and liver transplant hospitalizations with CCI>2 increased substantially over the study period

The demographics and comorbidity burden of hospitalization with heart transplant changed significantly over the study period. The mean age increased from 56.8 to 59.1 years; hospitalizations with black race increased from 5.4 to 16.1% and hospitalizations with high comorbidity burden (CCI>2) tripled from 27.4% to 68.8%. (Fig. 3 and Additional file 2: Table S1). The baseline characteristics of cardiac transplant hospitalizations complicated by AKI-D vs. those without are shown in Table 1. Hospitalizations with AKI-D were older (60.7 vs. 58.2 years) and more often male (76.4% vs. 72.6%; *p*<0.001) than those without. They also had worse co-morbidity scores (*p*<0.01) Finally, they exhibited significantly higher proportions of hypertension, liver disease, acute myocardial infarction, sepsis, cardiac catheterizations and mechanical ventilation. Interestingly, we identified a lower proportion of diabetes mellitus in AKI-D patients vs. those without. AKI-D patients were more likely to have a lower income status and more likely to be Medicare/Medicaid beneficiaries. With respect to hospital level characteristics, hospitalizations in large, urban-teaching hospitals were more likely to have AKI-D documented.

A series of sequential models to attempt to explain temporal increase in AKI-D trends is shown in Table 2. A univariable model including only calendar year demonstrated that the odds of AKI-D increased annually by approximately 5% (OR 1.05; 95% CI 1.02-1.09) between 2002 and 2013. Adjustment for patient demographics attenuated this to a 3% annual increase (adjusted OR 1.03; 95% CI 1.00-1.06). After adjustment for both demographics and concurrent comorbidities and procedures, the impact of calendar year was completely attenuated (adjusted OR 1.01, 95% CI 0.90-1.05;*p*=0.39). The significant comorbidities associated with increased temporal trend in AKI-D is shown in Table 3.

**Table 2:** Sequential Adjusted Models to Explain Temporal Trends of AKI Requiring Dialysis

	Unadjusted Odds Ratio/year (95% CI)	*P*	Adjusted Odds Ratio/year (95% CI) Model 1	*P*	Adjusted Odds Ratio/year (95% CI) Model 2	*P*
Heart transplant	1.05 (1.02 - 1.09)	<0.01	1.03 (1 - 1.06)	0.04	1.01 (0.9 - 1.05)	0.39
Liver transplant	1.06 (1.04 - 1.08)	<0.01	1.04 (1.01 - 1.06)	<0.01	1.024 (0.99 - 1.05)	0.09

Model 1: Adjusted for changes in age, sex and race

Model 2: Model 1 + Changes in comorbidities (HIV status, diabetes, hypertension, CKD, sepsis, heart failure, chronic liver diseases, liver cancer) and procedures (cardiac catheterizations and mechanical ventilation)

**Table 3:** Significant Comorbidities associated with Increased Temporal Trend of AKI-D in Heart and Liver Transplant Hospitalizations

	Heart Transplant		Liver Transplant	
	OR (95% CI)	*P*	OR (95% CI)	*P*
Year	1.01 (0.9 - 1.1)	0.39	1.02 (0.9 - 1.1)	0.09
Hypertension	1.93 (1.5 - 2.4)	<0.001	1.84 (1.5 - 1.1)	<0.001
Diabetes Mellitus	0.81 (0.6 - 0.9)	0.03	0.92 (0.8 - 0.0)	0.31
Chronic Kidney Disease	0.77 (0.5 - 1.0)	0.05	0.82 (0.6 - 0.0)	0.06
Sepsis	4.05 (3.1 - 5.1)	<0.001	3.25 (2.7 - 3.3)	<0.001
Primary Acute Heart Failure	2.38 (1.9 - 2.9)	<0.001	3.07 (2.5 - 3.3)	<0.001
Cardiac catheterization	0.53 (0.2 - 1.0)	0.06	0.64 (0.4 - 0.0)	0.04
Liver Disease	2.89 (2.1 - 3.9)	<0.001	-	-
Mechanical Ventilation	5.90 (4.5 - 7.7)	<0.001	5.40 (4.3 - 5.5)	<0.001

AKI-D occurring during hospitalizations in cardiac transplant recipients was associated with three-fold odds of mortality (adjusted OR 2.85; 95% CI 2.11-3.80) and two-fold odds of adverse discharge (adjusted OR 1.97; 95% CI 1.53-2.55) after adjusting for patient and hospital level confounders (Table 4).

**Table 4:** Adjusted Estimates of Impact of Acute Kidney Injury Requiring Dialysis on Mortality and Adverse Discharge

	Proportion of Non AKI-D vs. AKI-D	Adjusted Odds Ratio (95% Confidence Interval)	*P*
Mortality			
Heart transplant	2.11% vs. 16.94%	2.85 (2.11 - 3.8)	<0.01
Liver transplant	1.61% vs. 11.65%	2.00 (1.55- 2.59)	<0.01
Adverse Discharge			
Heart transplant	13.61% vs. 27.93%	1.97 (1.53-2.55)	<0.01
Liver transplant	13.38% vs. 34.21%	1.91 (1.57-2.30)	<0.01

### Liver transplant hospitalizations

The demographics and comorbidity burden of hospitalization with heart transplant changed significantly over the study period. The mean age increased from 53.2 to 67.5 years; hospitalizations with black race increased from 4.5 to 8.5% and hospitalizations with high comorbidity burden (CCI>2) doubled from 24.2 to 53%. (Fig. 3 and Additional file 2: Table S1). The baseline characteristics of liver transplant hospitalizations complicated by AKI-D vs. those without are shown in Table 1. Similar to cardiac transplant, hospitalizations with AKI-D were older and more male and with a higher proportion of African Americans/Hispanic compared to those without. They also tended to have worse co-morbidity scores. Finally, they exhibited significantly higher proportions of chronic comorbidities including diabetes mellitus, hypertension, liver disease, chronic kidney disease and liver/biliary cancer. They also had a higher proportion of acute comorbidities including acute myocardial infarction, sepsis, heart failure and mechanical ventilation. They also had lower income status and were more likely to be Medicare/Medicaid beneficiaries. With respect to hospital level characteristics, NRSOT hospitalizations in large, urban-teaching hospitals were more likely to have AKI-D documented.

Similar to cardiac transplant, a univariable model including only calendar year demonstrated that the odds of AKI-D increased annually by 6% (OR 1.06; 95% CI 1.04-1.08) between 2002 and 2013. Adjustment for patient demographics attenuated this to a 4% annual increase (adjusted OR 1.04; 95% CI 1.01-1.06). After adjustment for both demographics and concurrent comorbidities and procedures, the impact of calendar year was completely attenuated (adjusted OR 1.02, 95% CI 0.99-1.05;*p*=0.10). The significant comorbidities associated with increased temporal trend in AKI-D is shown in Table 3.

AKI-D occurring during hospitalizations in liver transplant recipients was associated with two-fold odds of mortality (adjusted OR 2.00; 95% CI 1.55-2.59) and two-fold odds of adverse discharge (adjusted OR 1.91; 95% CI 1.57-2.30) after adjusting for patient and hospital level confounders (Table 4).

## Discussion

In our analysis of nationally representative data, we demonstrate that the incidence of AKI-D in subsequent hospitalizations after receiving orthotopic cardiac and liver transplant has significantly increased from 2002–2013. We also show that temporal changes in demographics, comorbidities and procedures in part explain the increased yearly incidence. Finally AKI-D is significantly associated with an increased risk of mortality and adverse discharge among cardiac and liver transplant recipients.

Previous estimates of AKI and AKI-D in the immediate post transplantation period for orthotopic cardiac transplant range from 5–10% and for liver transplant range from 8–17% [10, 11, 20, 21], However, these studies were from a single year and/or center. In addition, they chose to focus on cross sections of cohorts rather than examining trends. Here, we chose to focus our analyses on AKI-D as our main outcome measure. This was largely due to prior publications emanating from administrative databases indicating that the specificity and sensitivity of transplant status and AKI-D codes are excellent. On the other hand, studies focusing on long-term renal sequelae after NRSOT have established relationship between post-operative AKI event and cumulative incidence of CKD over time [9].

We demonstrate that the incidence of AKI-D in subsequent hospitalizations following cardiac and liver transplantations increased over time and is tenfold higher than in the general population without NRSOT [3]. In addition, the temporal trend of AKI-D frequency has significantly increased over the study period; and changes in demographics, acute/chronic comorbidities and procedures are partly responsible for this rise. In other words, over time, as we are able to offer and achieve transplantation in patients with greater co-morbid disease burden, complications like AKI-D during subsequent acute care have increased. This raises the possibility that with improving graft/patient survival, in addition to transplant related risks, transplant recipients are also susceptible to demographic and comorbidity related risk factors, which may contribute to higher incidence of AKI-D over time [22] Considering, the growing evidence linking AKI and CKD, an increased incidence of AKI-D could be in part responsible for the increased chronic renal dysfunction seen in solid organ transplant recipients [23].

We also demonstrate that risks for AKI-D differ in cardiac and liver transplant hospitalizations. For example, CKD was associated with AKI-D in liver but not cardiac transplant hospitalizations. It is plausible that under-recognition of renal dysfunction due to inability of serum creatinine to accurately estimate renal function in in liver transplant settings may lead to “CKD” diagnosis to represent more severe kidney disease than other settings [24]. However, this study does provide insight into several major risk factors, which could be used to risk-stratify those recipients who may be at a higher risk of developing AKI-D in hospitalizations after their transplant surgeries.

Our analysis has limitations. NIS registries do not provide patient level data, including laboratory data or medication data. Thus we could not ascertain baseline CKD stage or the contribution of medications to the development of AKI-D. Also since the data are completely de-identified, we cannot delineate the risk of recurrent episodes of AKI-D or recurrent hospitalizations in the same patients over time. Also, we chose to focus on AKI-D as our main outcome measure. This was largely due to prior publications emanating from administrative databases indicating that the specificity and sensitivity of transplant status and AKI-D codes are excellent [25]. Although we cannot exclude more liberal use of acute dialysis over time [26], in the general population both dialysis-requiring and laboratory-defined AKI have been increasing in incidence [6]. We also could not distinguish between intermittent and continuous dialysis therapies. We are also unable to account for unmeasured confounders that could be associated with increasing incidence of AKI –D, such as degree of severity of acute illness, although we use the Charlson comorbidity score to adjust for comorbid disease burden, which is the present standard utilized when analyzing administrative databases. Also, our study design did not include immediate post-operative dialysis requirement or those who could have died before being considered at risk for receiving dialysis during subsequent hospitalizations. We expect this proportion to be relatively small [27]. More importantly, we did not include immediate post transplant period, as “new dialysis” in immediate perioperative period especially for liver transplant may not necessarily be employed for AKI, but could be for pre-operative CKD/AKI, as well as other fluid/electrolyte and volume related indications. Finally, we could not ascertain the indication for dialysis since we lacked patient level, granular data Thus, although our analysis provides a conservative estimate of the rising burden of AKI-D in transplant-associated hospitalizations; we believe that it is a more accurate reflection of dialysis requirement for true kidney injury.

## Conclusions

In summary, this nationwide sample provides secular, temporal trends of AKI-D in recipients of orthotopic heart and liver transplant hospitalizations. With an established connection between post-transplant AKI and CKD, it is important to study the growing burden of those who develop AKI and are at risk of CKD after transplant. Also, the analysis indicates that the rising trends in AKI-D requirement is in part explained by demographics and co-morbid disease burden of transplant recipients over time. Said in another way, as we get better at caring for older and sicker patients to survive with transplantation, we need to recognize the medical and healthcare economic burden associated with complex complications such as AKI-D. As was shown, AKI-D in transplant recipients is associated with higher mortality and adverse discharge. Future studies need to delineate risk factors and outcomes of milder forms of AKI by utilizing patient-level laboratory information and medication information. Additionally, we need to develop strategies to reduce the risk of AKI in solid organ transplant recipients to improve health outcomes.

## Additional files

Additional file 1: Figure S1. Sequential derivation of the study population from NIS 2002-2013 database. This figure demonstrates the derivation of the study population of interest from the larger Nationwide Inpatient Sample database. (PPTX 57 kb)

Additional file 2: Table S1. Changes in Demographics and Comorbidities for Heart and Liver Transplant Hospitalizations from 2002-2013. This table shows the changes in demographics and Charlson comorbidity index for Heart and Liver Transplant Hospitalizations from 2002-2013. (DOCX 145 kb)

## Abbreviations

AKI: Acute kidney injury
AKI-D: AKI requiring dialysis
CCI: Charlson co-morbidity index
CCS: Clinical Classification Software
ESRD: End-stage renal disease
HCUP: Healthcare Cost and Utilization Project
ICD-9-CM: International Classification of Diseases, Ninth Revision, Clinical Modification
NIS: Nationwide Inpatient Sample
NRSOT: Non-renal solid organ transplantation
