# Supplementary material for: Temporal trends of dialysis requiring acute kidney injury after orthotopic cardiac and liver transplant hospitalizations
Source: BMC Nephrol. 2017 Jul 19;18:244. doi: 10.1186/s12882-017-0657-8 (PMC5516358; doi:10.1186/s12882-017-0657-8)
Supplement: Supplementary file 1 — Sequential derivation of the study population from NIS 2002-2013 database. This figure demonstrates the derivation of the study population of interest from the larger Nationwide Inpatient Sample database. (PPTX 57 kb) [file 12882_2017_657_MOESM1_ESM.pptx]

## Slide 1
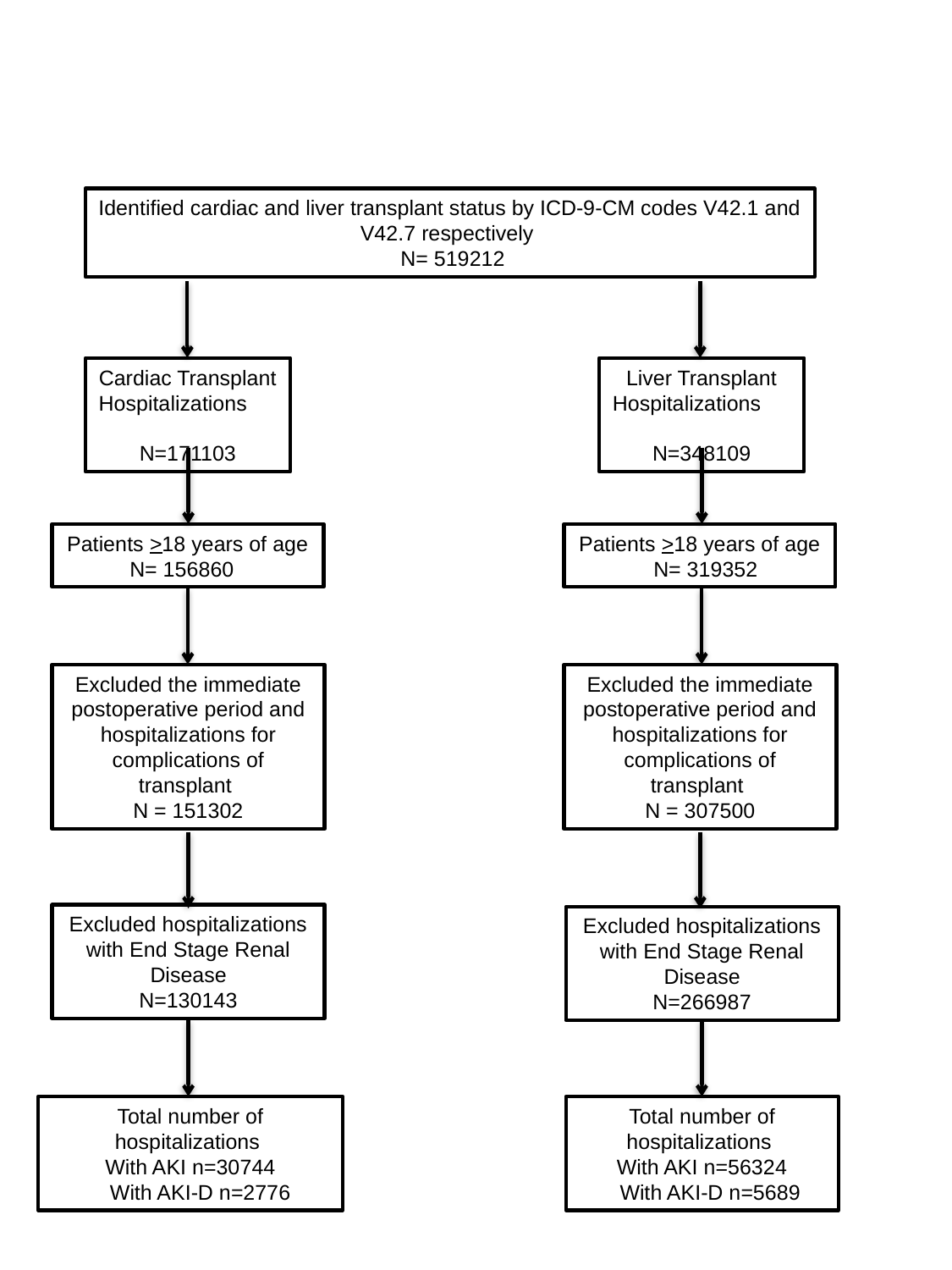

Identified cardiac and liver transplant status by ICD-9-CM codes V42.1 and V42.7 respectively
 N= 519212
Cardiac Transplant Hospitalizations N=171103
Liver Transplant Hospitalizations N=348109
Patients >18 years of age
 N= 156860
Patients >18 years of age
 N= 319352
Excluded the immediate postoperative period and hospitalizations for complications of transplant
N = 151302
Excluded the immediate postoperative period and hospitalizations for complications of transplant
N = 307500
Excluded hospitalizations with End Stage Renal Disease
N=130143
Excluded hospitalizations with End Stage Renal Disease
N=266987
Total number of hospitalizations
With AKI n=30744
 With AKI-D n=2776
Total number of hospitalizations
With AKI n=56324
 With AKI-D n=5689
