# Supplementary material for: Temporal trends of dialysis requiring acute kidney injury after orthotopic cardiac and liver transplant hospitalizations
Source: BMC Nephrol. 2017 Jul 19;18:244. doi: 10.1186/s12882-017-0657-8 (PMC5516358; doi:10.1186/s12882-017-0657-8)
Supplement: Supplementary file 2 — Changes in Demographics and Comorbidities for Heart and Liver Transplant Hospitalizations from 2002-2013. This table shows the changes in demographics and Charlson comorbidity index for Heart and Liver Transplant Hospitalizations from 2002-2013. (DOCX 145 kb) [file 12882_2017_657_MOESM2_ESM.docx]

**Supplementary Table 1.** Changes in Demographics and Comorbidities for Heart and Liver Transplant Hospitalizations from 2002-2013

| **Heart Transplant** | | | | | | | | | | | | | |
| --- | --- | --- | --- | --- | --- | --- | --- | --- | --- | --- | --- | --- | --- |
|  | 2002 | 2003 | 2004 | 2005 | 2006 | 2007 | 2008 | 2009 | 2010 | 2011 | 2012 | 2013 | P |
| Age Mean (SE) | 56.82 (0.50) | 56.63 (0.66) | 58.64 (0.59) | 58.15 (0.52) | 57.36 (0.66) | 58.36 (0.79) | 57.67 (0.60) | 58.24 (0.46) | 59.03 (0.59) | 59.64 (0.56) | 58.51 (0.40) | 59.06 (0.38) | <0.001 |
| Race (%) | | | | | | | | | | | | | <0.001 |
| White | 55.4 | 63.7 | 63.6 | 59.0 | 60.0 | 59.6 | 59.0 | 60.2 | 71.5 | 70.7 | 68.5 | 65.9 |  |
| Black | 5.4 | 12.3 | 9.4 | 6.5 | 10.0 | 12.4 | 8.6 | 13.0 | 11.8 | 13.5 | 15.4 | 16.1 |  |
| Hispanic | 2.7 | 5.0 | 5.3 | 4.2 | 3.8 | 5.6 | 6.5 | 5.3 | 6.7 | 5.0 | 6.6 | 6.4 |  |
| Others | 1.0 | 3.1 | 2.2 | 1.9 | 2.9 | 4.0 | 5.4 | 4.2 | 3.3 | 4.2 | 4.4 | 4.5 |  |
| Missing | 35.5 | 15.9 | 19.6 | 28.5 | 23.3 | 18.4 | 20.5 | 17.3 | 6.7 | 6.6 | 5.0 | 7.3 |  |
| Charlson Comorbidity Index (%) | | | | | | | | | | | | | <0.001 |
| 0 | 43.3 | 41.1 | 40.7 | 34.4 | 29.5 | 22.0 | 25.1 | 21.6 | 19.9 | 19.0 | 19.7 | 16.7 |  |
| 1 | 29.3 | 31.7 | 30.7 | 32.1 | 25.2 | 20.0 | 18.5 | 18.9 | 16.8 | 17.4 | 17.3 | 14.6 |  |
| 2 | 27.4 | 27.2 | 28.7 | 33.5 | 45.3 | 58.0 | 56.4 | 59.5 | 63.3 | 63.7 | 63.1 | 68.8 |  |
| **Liver Transplant** | | | | | | | | | | | | | |
| Age Mean (SE) | 53.20 (0.37) | 53.76 (0.37) | 54.45 (0.40) | 54.28 (0.44) | 55.03 (0.42) | 55.44 (0.36) | 55.45 (0.40) | 56.17 (0.35) | 55.83 (0.36) | 57.48 (0.37) | 57.04 (0.22) | 57.46 (0.25) | <0.001 |
| Race (%) | | | | | | | | | | | | | <0.001 |
| White | 53.2 | 60.5 | 60.0 | 56.1 | 52.3 | 50.1 | 59.7 | 62.1 | 69.0 | 68.2 | 69.4 | 67.5 |  |
| Black | 4.5 | 6.7 | 6.6 | 3.7 | 4.8 | 5.6 | 5.3 | 5.6 | 8.7 | 8.3 | 7.8 | 8.5 |  |
| Hispanic | 9.1 | 10.5 | 11.4 | 11.7 | 7.8 | 11.0 | 11.0 | 11.4 | 9.8 | 11.9 | 10.7 | 12.1 |  |
| Others | 4.1 | 5.2 | 4.2 | 5.6 | 3.8 | 6.1 | 5.6 | 5.6 | 5.0 | 4.7 | 6.7 | 5.8 |  |
| Missing | 29.1 | 17.1 | 17.9 | 23.0 | 31.2 | 27.2 | 18.3 | 15.4 | 7.6 | 6.9 | 5.5 | 6.1 |  |
| Charlson Comorbidity Index (%) | | | | | | | | | | | | | <0.001 |
| 0 | 46.9 | 46.4 | 43.5 | 42.6 | 37.7 | 33.3 | 35.5 | 31.8 | 29.0 | 27.0 | 26.9 | 27.1 |  |
| 1 | 28.9 | 28.9 | 30.8 | 29.6 | 25.1 | 23.3 | 21.9 | 21.3 | 22.7 | 20.7 | 21.0 | 20.0 |  |
| 2 | 24.2 | 24.8 | 25.7 | 27.8 | 37.2 | 43.4 | 42.6 | 46.9 | 48.3 | 52.2 | 52.1 | 53.0 |  |

Se=Standard Error

All figures shown are percentages from the total number of hospitalizations with heart or liver transplantation.
